# Fine Mapping of a GWAS-Derived Obesity Candidate Region on Chromosome 16p11.2

**DOI:** 10.1371/journal.pone.0125660

**Published:** 2015-05-08

**Authors:** Anna-Lena Volckmar, Jie-Yun Song, Ivonne Jarick, Carolin Pütter, Maria Göbel, Lucie Horn, Christoph Struve, Katharina Haas, Nadja Knoll, Harald Grallert, Thomas Illig, Thomas Reinehr, Hai-Jun Wang, Johannes Hebebrand, Anke Hinney

**Affiliations:** 1 Department of Child and Adolescent Psychiatry, University Hospital Essen, University of Duisburg-Essen, Essen, Germany; 2 Institute of Child and Adolescent Health, School of Public Health, Peking University, Beijing, China; 3 Institute of Medical Biometry and Epidemiology, University of Marburg, Marburg, Germany; 4 Institute of Epidemiology, Helmholtz-Zentrum Munich, Munich, Germany; 5 Hannover Unified Biobank, Hannover Medical School, Hannover, Germany; 6 Institute for Human Genetics, Hannover Medical School, Hannover, Germany; 7 Vestische Hospital for Clinic of Children and Adolescents Medicine, Datteln, University of Witten/Herdecke, Witten, Germany; McMaster University, CANADA

## Abstract

**Introduction:**

Large-scale genome-wide association studies (GWASs) have identified 97 chromosomal loci associated with increased body mass index in population-based studies on adults. One of these SNPs, rs7359397, tags a large region (approx. 1MB) with high linkage disequilibrium (r²>0.7), which comprises five genes (*SH2B1*, *APOBR*, sulfotransferases: *SULT1A1* and *SULT1A2*, *TUFM*). We had previously described a rare mutation in *SH2B1* solely identified in extremely obese individuals but not in lean controls.

**Methods:**

The coding regions of the genes *APOBR*, *SULT1A1*, *SULT1A2*, and *TUFM* were screened for mutations (dHPLC, SSCP, Sanger re-sequencing) in 95 extremely obese children and adolescents. Detected non-synonymous variants were genotyped (TaqMan SNP Genotyping, MALDI TOF, PCR-RFLP) in independent large study groups (up to 3,210 extremely obese/overweight cases, 485 lean controls and 615 obesity trios). *In silico* tools were used for the prediction of potential functional effects of detected variants.

**Results:**

Except for *TUFM* we detected non-synonymous variants in all screened genes. Two polymorphisms rs180743 (*APOBR* p.Pro428Ala) and rs3833080 (*APOBR* p.Gly369_Asp370del9) showed nominal association to (extreme) obesity (uncorrected p = 0.003 and p = 0.002, respectively). *In silico* analyses predicted a functional implication for rs180743 (*APOBR* p.Pro428Ala). Both *APOBR* variants are located in the repetitive region with unknown function.

**Conclusion:**

Variants in *APOBR* contributed as strongly as variants in *SH2B1* to the association with extreme obesity in the chromosomal region chr16p11.2. *In silico* analyses implied no functional effect of several of the detected variants. Further *in vitro* or *in vivo* analyses on the functional implications of the obesity associated variants are warranted.

## Introduction

The largest GWAS study on BMI including a total of 339,224 individuals identified 97 genetic loci associated with increased BMI [1]. One chromosomal region (chr16p11.2) is tagged by two lead SNPs separated by more than 500 kb. One of the SNPs (3888190) is located near *ATP2A1* (ATPase, Ca++ transporting, cardiac muscle, fast twitch 1 gene) and *SH2B1* (Src-homology 2B adaptor protein 1 gene), the other signal (rs2650492) is near *SBK1* (SH3 domain binding kinase 1 gene) and *APOBR* (apolipoprotein B receptor gene). Both tag a large region with high linkage disequilibrium (LD) which has solidly been replicated for obesity and BMI [2–14]. Besides the GWAS findings, a large deletion in the same chromosomal region 16p11.2 was associated with obesity [15–20], developmental delay and autism [15–19]. The reciprocal duplication of the same chromosomal region is associated with reduced BMI, developmental delay and schizophrenia [21].

The most plausible obesity gene in the region is *SH2B1*. SH2B1 is a mediator of energy homeostasis and increases leptin and insulin potency in downstream signaling pathways [22]. *Sh2b1* knockout mice are obese, hyperphagic and exhibit traits of the metabolic syndrome like hyperlipidemia, leptin resistance, hyperglycemia, and insulin resistance [23]. In *SH2B1* we detected a rare mutation solely in obese individuals; additionally we replicated the obesity association of the GWAS SNP rs7498665 (*SH2B1*: p.Thr484Ala; [14]). Another group also described *SH2B1* mutations in extremely obese children with insulin resistance [24, 25]. As the infrequent mutations cannot explain the genome-wide association signal and so far, no functional effect of the frequent SNP has been detected [14, 25], we analysed additional promising obesity candidate genes in the same chromosomal region.

Lead SNPs in GWAS can tag large regions of high linkage disequilibrium (LD) which can comprise one to several genes/variants that are relevant for the analyzed phenotype [26]. For the region on chr16p11.2, Speliotes et al. [2] listed non-synonymous SNPs in adjacent genes (*SH2B1*, *APOBR*, *SULT1A2*) in high LD (r^2^>0.75) with the lead SNP rs7359397 ([2], see supplementary material). Additionally for a total of four adjacent genes, involvement in weight regulation seems likely because they are either (a) biological candidates (*SH2B1*), or differentially expressed in adipose tissue between general population and patients who underwent bariatric surgery (*SH2B1*, *SULT1A1*, *SULT1A2*, *TUFM*; [1]). Further fine mapping of the chromosomal region for causal variants that contribute to the obesity association has not yet been undergone.

The *APOBR* gene encodes a macrophage receptor that regulates fat and vitamin uptake into cells [27, 28]. Non-synonymous variants (rs180743: p.Pro428Ala and rs3833080: p.Gly369_Asp370del9) in *APOBR* are associated with hypercholesterolemia [29]. Mice fed a high fat diet until they became obese showed increased expression of *APOBR* and hence increased lipid intake in macrophages of the adipose tissue [28]. This is mediated by the transcription factors PPARα, PPARβ/δ, PPARγ and the PPAR-RXR transcriptional complex [30, 31]. In normal weight humans, a single meal with high fat content (72% of the total energy of the meal) increased both *APOBR* expression and lipid uptake in monocytes [32]. High blood lipid levels differentially regulate *APOBR* expression in human postprandial monocytes and macrophages and lead to foam cell formation [33].

The sulfotransferase genes *SULT1A1* and *SULT1A2* are located close to each other on chr16p11.2. Both proteins sulfonate hormones like estrogens, estrogenic alkylphenols, 17-β-estradiol and several androgens, so that the hormones can be excreted [34]. Obesity is associated with increased levels of 17-β-estradiol, estron and estron sulfate which are substrates of SULT1A2 [35–37]. Childhood obesity is associated with an increased risk of adult obesity and Type 2 Diabetes mellitus [38]. Association of a non-synonymous SNP (rs141581853: *SULT1A1* p.Arg213His) with obesity but not hypertension had been described [39]. The regulation of *SULT1A1* expression in diet induced obesity (DIO) rats in adipose tissue and liver was dependent on the dietary fat content [40].

*TUFM* encodes a transcription factor for mitochondrial gene expression [41]. (1) Exclusive maternal inheritance of mitochondria and mitochondrial DNA, (2) stronger correlation with maternal than paternal BMI [42] and (3) the relevance of mitochondria for energy metabolism indicate that genes involved in mitochondrial function are relevant candidate genes for weight regulation. *TUFM* expression is up regulated in DIO rats on a high fat diet [40]. In human cultured hippocampal neurons, BDNF stimulation down regulated the expression of *TUFM* [43].

In order to fine map the chromosomal region chr16p11.2 for further obesity associated variants, we screened the coding regions of *APOBR*, *SULT1A1*, *SULT1A2*, and *TUFM* for variants in 95 extremely obese children and adolescents. Most of these individuals were enriched for the likely presence of mutations in high LD with the original obesity association signal [2, 14]. Our focus was the detection of common to infrequent variants (MAF > 0.01) which affect the protein sequence. Previously it was shown, for instance for the MC4R, that GWAS results point to genes in which functionally relevant mutations are found more frequently in cases than in controls [44]. These infrequent mutations might also have a major gene effect. Although synthetic association does not seem to be a frequent mechanism [45], GWAS results and mutation screens frequently depict the same genes. Subsequently, we confirmed the detected non-synonymous variants in independent study groups.

## Material and Methods

### Study groups

An overview of the study groups can be found in Table 1 (see also [14]), details of recruitment have been described previously [46, 47]. We included children and adolescents (mean age = 13.25 ± 3.26 years) with a BMI above the 97th BMI percentile. Written informed consent was given by all participants and in case of minors by their parents. These studies were approved by the Ethics Committees of the respective Universities (Marburg: ‘Ethik-Kommission des Fachbereichs Medizin der Philipps-Universität Marburg’: ethic commission of the Medical Faculty of the Philipps-University Marburg, Duisburg-Essen: ‘Ethik-Kommission der Medizinischen Fakultät der Universität Duisburg-Essen’: ethic commission of the Medical Faculty of the University of Duisburg-Essen) and were performed in accordance with the Declaration of Helsinki.

**Table 1:** Phenotypic description of analyzed study groups.

			% male	age	BMI	BMI SDS
Sample	Status	n	[%]	[mean ± SD]	[mean ± SD]	[mean ± SD]
**Screening Sample**	Cases	95	48.89	13.25 ± 3.26	31.79 ± 5.13	4.06 ± 5.13
**Family-based GWAS for obesity**	Cases	615	45.11	13.44 ± 3.02	32.02 ± 5.82	4.23 ± 1.96
	Parents	1,230	50.00	42.54 ± 6.02	30.28 ± 6.33	1.65 ± 1.84
**DAPOC**	Cases	1,383	44.25	10.79 ± 2.84	27.82 ± 5.13	3.11 ± 1.58
**Case-control GWAS for obesity**	Cases	453	42.60	14.37 ± 3.75	33.15 ± 6.68	4.51 ± 2.15
	Controls	435	39.08	26.08 ± 5.75	18.09 ± 1.14	-1.45 ± 0.35

Mutation screen sample: part of the family-based and the case-control GWAS samples’ cases (90 extremely obese index patients from the 705 family-based GWAS trios; 5 extremely obese patients from the case-control GWAS). Family-based GWAS sample: 615 index patients with extreme obesity and their biological parents; independent of initial screening sample [14, 46]. The 355 trios used for association analysis was part of this sample but did not deviate significantly from the description given for the overall sample. Case-control GWAS sample: GWAS of extremely obese children and adolescents in comparison to lean, adult controls; independent of initial screening sample [14, 47]. DAPOC: Datteln Paediatric Obese Cohort: Sample of overweight and obese children and adolescents; independent of initial screening sample [54].

### Mutation screen

The selection of extremely obese individuals for the mutation screen was based on genotypes at SNP rs2008514 (proxy of rs7359397; [2]) in the chromosomal region 16p11.2. In total, we analyzed 95 extremely obese individuals, 90 of whom were likely enriched for the presence of infrequent mutations at chr16p11.2 that contribute to the association signal of rs2008514. These extremely obese patients (offspring) from the family-based GWAS sample were homozygous for the obesity risk allele T at rs2008514 and had at least one heterozygous parent, thus substantially contributing to the observed over-transmission of the rs2008514 T-allele in our previous study [20]. The other five individuals harbor a deletion on chr16p11.2 that does not include the genes which were screened for mutations here. The 95 individuals were screened for mutations in *APOBR* (NM ID: 55911, chr16:28,505,970–28,510,291), *SULT1A1* (NM ID: 6817, chr16:28,617,142–28,620,176), *SULT1A2* (NM ID: 6799, chr16:28,603,349–28,607,251) and *TUFM* (NM ID: 7284, chr16:28,854,296–28,857,590, positions given for GRCh37/hg19). All primers can be found in (S3 Table).

Depending on the size of the screened fragment, one of the following two methods was used for the mutation screen of the coding region of each gene as described previously [14, 48]: We used single stranded conformation polymorphism analyses for PCR amplicons up to 300bp [49] or denaturing high-performance liquid chromatography for PCR amplicons up to 600bp [50, 51]. Using these fragment sizes both methods achieve a high sensitivity (below 5% error rate [49, 50]) which is very well compatible with Sanger sequencing [52, 53]. All PCR amplicons with dHPLC/SSCP patterns deviant from the wild-type pattern were re-sequenced as described previously [14]. At least two experienced individuals independently assigned the deviant patterns; discrepancies were solved either by reaching consensus or by re-screening.

### Genotyping

**Fig 1:**
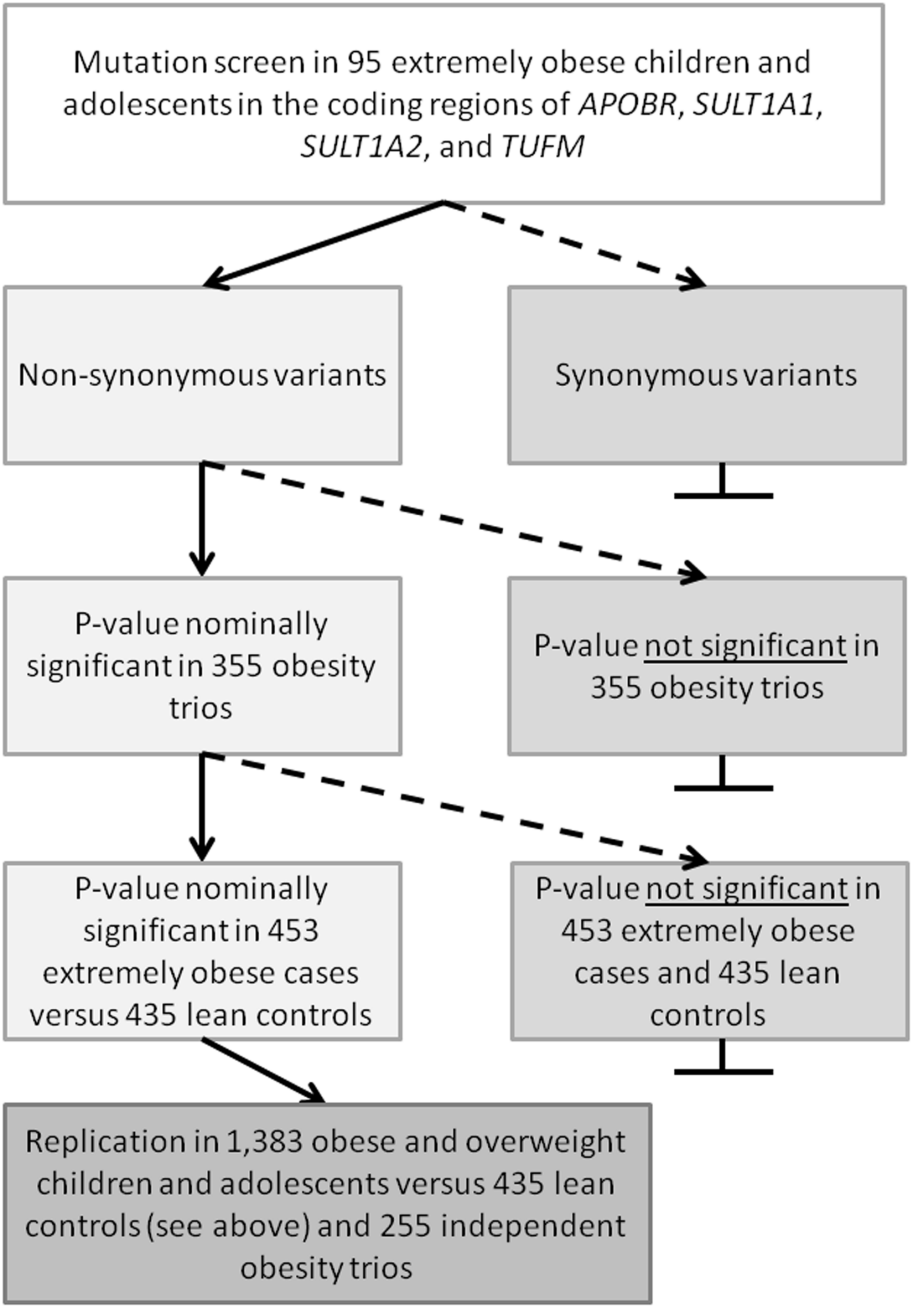
Decision tree for genotyping of variants detected in the initial screening collective of 95 extremely obese children and adolescents.

The non-synonymous variants identified in the mutation screens in *SULT1A1*, and *SULT1A2* were genotyped in 355 obesity families [46] by MALDI TOF, RFLP and tetra ARMS PCR (Fig 1). The missense variants in *APOBR* (rs180743: p.Pro428Ala; rs3833080: p. Gly369_Asp370del9; rs368546180: p.Thr321_Gly329del9) were genotyped in the following independent study groups by either gel electrophoresis of PCR products (for deletions and insertions) or TaqMan assay (detailed information can be obtained from the authors): 615 obesity trios (extremely obese child or adolescent with both biological parents; [20]) and the case-control GWAS study groups (453 extremely obese cases and 435 lean controls described in [46, 47]; see above) and 1,383 obese and overweight children and adolescents (Datteln Paediatric Obese Cohort [54]). At least two experienced individuals independently assigned the genotypes; discrepancies were solved either by reaching consensus or by re-genotyping. In case of the trios, Mendelian inheritance was checked. For the other study groups, Hardy Weinberg equilibrium was assessed and fulfilled. All enzymes and protocols can be obtained from the authors.

### Statistics

For association studies in the above-mentioned 453 cases and 435 controls Fisher’s exact test (allelic association) was calculated with PLINK [55] adjusted for sex and age of the individuals. In the 615 trios, an asymptotic, 2-tailed p-value for the transmission disequilibrium test (TDT [56]) was calculated with PLINK. The initial screening sample was excluded from the further analyses. All p-values are asymptotic, two-sided and not corrected for multiple testing unless stated otherwise. Additionally to univariate analysis, we conducted a joint analysis of the significant SNPs to reveal if these SNPs descend from the same signal. This was done with R 3.1.0 [57] without any further adjustment.

### Functional *in silico* analyzes

All detected variants were analyzed for loss or gain of cryptic splice sites (ESEfinder [58], ESRSearch [59], RESCUE_ESE [60]), and transcription factor binding sites (TFSearch [61], Consite [62]). Prediction of an impact of an amino acid exchange on structure and function was performed by PANTHER [63], PolyPhen-2 [64], SNAP [65], PMUT [66], and MutationTaster [67]. For the InDel variants, PROVEAN [68] was used for functional prediction.

## Results

### APOBR

The mutation screen of the coding region of the *APOBR* in 95 extremely obese children and adolescents revealed 13 sequence variants (rs74949322, rs151233, rs149271, rs3833080, rs368546180, rs180743, rs180744, rs151174, rs40831, rs61738759, rs200751685, rs40833, and rs142786317; S1 Table). Three of these affect the amino acid sequence (rs368546180: p.Thr321_Gly329del9, rs3833080: p.Gly369_Asp370del9, and rs180743: p.Pro428Ala; Table 2).

**Table 2:** In silico functional prediction of detected non-synonymous variants in chr16p11.2 (screened genes APOBR, SH2B1, SULT1A1, and SULT1A2).

			PolyPhen-2		SNAP		SIFT			Mutation Taster		PANTHER	PROVEAN	
Gene	Amino acid changes *rs-number*	DNA position	*Delta_Score*	*Prediction*	*Prediction*	*Score*	*Prediction*		*Score*	*Prediction*	*Score*	*Prediction*	*Prediction*	*Score*
***APOBR***	Thr321_Gly329 del9 *rs368546180*	c.933_934delA CAGCCTCAG GCGGGGAGG AGGCTGAA	NA		NA		NA			Polymorphism	0.71	NA	deleterious	-3.0
	Gly369_Asp370 del9 *rs3833080*	c.1035_1036del GGGACAGCC TCAGGAGGG GAGGAGGCC	NA		NA		NA			Polymorphism	1	NA	neutral	-2.1
	Pro428Ala *rs180743*	c.1282C>G	0.600	possibly damaging	neutral	0.73	deleterious	0.78		Polymorphism	1	neutral	NA	
***SH2B1***	Thr174Asn *rs181294111*	g.2749C>A	0.000	benign	neutral	0.53	neutral	0.31		Polymorphism	1	neutral	NA	
	Thr484Ala *rs7498665*	g.8164A>G	0.219	benign	neutral	0.85	neutral	0.93		Polymorphism	1	neutral	NA	
	Thr656Ile Pro675Ser *rs369858622*	9483C>T	0.107	benign	not neutral	0.63	neutral	0.1		Polymorphism	0.96	NA	NA	
			0.038	benign	neutral	0.53	neutral	0.43		Polymorphism	0.96	NA	NA	
***SULT1A2***	Ile7Thr *rs1136703*	c.20T/C	0.000	benign	neutral	0.06	neutral	0.68		Polymorphism	1	neutral	NA	
	Pro19Leu *rs10797300*	c.56C/T	1.000	probably damaging	neutral	0.41	neutral	0.39		Polymorphism	0.99	deleterious	NA	
	Ser44Asn *rs145008170*	c.131G/A	0.021	benign	non-neutral	0.14	deleterious	0.03		Polymorphism	0.97	neutral	NA	
	Tyr62Phe *rs4987024*	c.185A/T	0.999	probably damaging	neutral	0.06	neutral	0.16		Polymorphism	0.99	NA	NA	
	Ala164Val *rs142241142*	c.491C/T	0.006	benign	neutral	0.60	neutral	0.21		Polymorphism	1	neutral	NA	
	Asn235Ile *rs1059491*	c.704A/C	1.000	probably damaging	non-neutral	0.81	deleterious	0		Polymorphism	0.04	NA	NA	

non-synonymous variants were not detected in *TUFM*; NA: not available. Data on *SH2B1* are obtained from our previous publication on a mutation screen in *SH2B1* [14] and displayed here to give a full overview on the mutations detected in the chromosomal region chr16p11.2 in the 95 screened obese individuals.

The common deletion rs3833080 (c.1036_1062del27; S1 Data) was present in each individual of the initial screen either hetero- or homozygously. The variant is located in the repeat region of *APOBR* with no predicted function and deletes one full repeat [GlyGlyGluGluAlaGluThrAlaSer] of the amino acid sequence [27]. *In silico* prediction for rs3833080 implicated no functional effect (Table 2).

The non-synonymous, non-conservative SNP rs180743: p.Pro428Ala is located closely to the deletion rs3833080 which is in high LD with the variant (r^2^ = 0.98). Due to the high LD with rs2008514, both variants were carried, at least heterozygously, in all individuals of the initial screen. Functional *in silico* prediction for the risk allele is variable, although the SNP is located in a conserved position (conservation 66% among 29 species, ENSEMBL). SIFT and PolyPhen2 rated this SNP as deleterious, while PANTHER, SNAP and PMUT rated it as neutral (Table 2). Although the overall prediction is “Polymorphism”, Mutation Taster predicted the introduction of a new splice site, thereby disruption of a glutamate-rich region and potential loss of a phosphoserine domain (Table 2).

The deletion rs368546180 with a length of 27bp (c.933_934insdel27; p.Ala328_Gly329) results in an in frame shortened amino acid sequence of the repeat region of *APOBR* with no predicted function [27]. The *in silico* prediction of the variant is ambiguous (Mutation Taster “Polymorphism”, SIFT “neutral”, PROVEAN “deleterious”; Table 2). It was identified once heterozygously in an extremely obese child. The female mutation carrier (height 147 cm, weight 49 kg, BMI 22.68 kg/m^2^, BMI SDS 1.44, age 10.5 years, 93^rd^ age and sex specific BMI percentile) inherited the mutation from her obese mother (BMI 39.06 kg/m^2^) while the overweight (BMI 25.65 kg/m^2^) father did not harbor the deletion. The girl also homozygously carried the risk alleles (minor alleles) at rs180743: p.Pro428Ala and rs3833080: p.Gly369_Asp370del9.

Case-control association analyses based on 1,873 extremely obese cases and 435 lean controls were performed for the two frequent, coding variants rs180743: p.Pro428Ala and rs3833080: p.Gly369_Asp370del9 and the second infrequent deletion rs368546180: p.Thr321_Gly329del9. The *APOBR* rs180743 G-allele was nominally associated with obesity (odds ratio (OR) per allele = 1.27; 95% confidence interval (CI): 1.09–1.47, p = 0.002, see Table 3). Similarly, the deletion allele of rs3833080 was nominally associated with obesity (OR = 1.25 per allele; 95%CI: 1.08–1.45, p = 0.003). Family-based association studies (based on 615 obesity trios) for rs3833080 and rs180743 confirmed these associations (Table 3). While genotyping rs3833080 we also observed the insertion allele (p.Gly369_Asp370insGluGluAlaGlyThrAlaSerGlyGly), with a much lower frequency and exclusively in (extremely) obese cases (minor allele frequency of 0.001 in 2,540 cases) but not in 481 lean or normal weight controls. The second deletion rs368546180: p.Thr321_Gly329del9 was only observed once in all screened individuals. Consequently, association analysis could not be performed (Table 3).

**Table 3:** Association analyses of polymorphism of detected non-synonymous variants in chr16p11.2 (screened genes APOBR, SH2B1, SULT1A1, and SULT1A2) in extremely obese children and adolescents and lean controls.

			Alleles (% MAF^a^)		Effect allele frequencies [%]					p-value (Bonferroni corrected)		
Gene	Position / rs-Number	Amino acid change	Minor	other	Index	Parents	Cases	Controls	Mendelian error rate [%]	Trios^b^	Cases & Controls^c^	OR 95% CI
***APOBR***	rs368546180*	Thr321_Gly329del9	del9 (0.79)	-	NA	NA	NA	NA	NA	NA	NA	NA
	rs3833080	Gly369_Asp370del9	del9 (31.03)	-	46.06	43.36	43.67	37.97	0.01	**0.004 (0.048)**	**0.002 (0.024)**	1.27 (1.09–1.47)
	rs180743	Pro428Ala	G (38.96)	C	45.53	46.14	43.68	38.26	1.70	**0.003 (0.036)**	**0.003 (0.036)**	1.25 (1.08–1.45)
***SH2B1***	rs181294111	Thr174Asn	A (0.05)	C	0.00	0.00	0.00	0.00	0.00	0.317	1	NA
	rs7498665	Thr484Ala	G (38.14)	A	56.01	58.38	40.52	35.63	0.00	**0.009** (0.108)	**0.001 (0.012)**	1.31 (1.13–1.52)
	rs369858622	Thr656Ile Pro675Ser	T (0.01)	C	0.00	0.00	0.00	0.00	0.00	1	1	NA
***SULT1A2***	rs4149403	Ile7Thr	C (NA)	T	57.14	56.65	NA	NA	0.00	0.634	NA	NA
	rs10797300	Pro19Leu	T (13.85)	C	11.90	11.71	NA	NA	0.29	0.752	NA	NA
	rs145008170	Ser44Asn	NA (0.04)	NA	-	-	NA	NA	0.00	0.157	NA	NA
	rs4987024	Tyr62Phe	A (0.80)	T	98.45	98.37	NA	NA	0.00	0.670	NA	NA
	rs142241142	Ala164Val	A (NA)	G	0.85	0.57	NA	NA	0.00	0.157	NA	NA
	rs1059491	Asn235Thr	A (36.08)	C	96.49	93.69	NA	NA	**6.78**	NA	NA	NA

^a^ Minor allele frequencies were taken from the European cohort of the exome variant server (http://evs.gs.washington.edu/EVS/).

^b^ 355 family based trios (extremely obese index patient with both biological parents), transmission disequilibrium test.

^c^ 423 underweight or normal weight adults and 1,873 extremely obese children and adolescents, Fisher’s exact test.

NA: not available. Data on *SH2B1* are obtained from our previous publication on a mutation screen in *SH2B1* [14] and displayed here to give a full overview on the mutations detected in the chromosomal region chr16p11.2 in the 95 screened individuals. The effect allele frequencies refer to the frequencies of the over-transmitted alleles in either the indexes or the parents. In case the variant was mono allelic in the analyzed sample (rs145008170), effect alleles could not be determined. For rs1059491, the p-value is not reported as the variant displayed a high Mendelian error rate (marked in bold). The high sequence similarity of *SULT1A1* and *SULT1A2* probably lead to congruent genotyping of the same variant Asn235Thr (rs1059491 and rs35728980, respectively) in both genes at the same time, despite choosing probes that were unique in the human genome (http://genome.ucsc.edu/).

*Variant rs368546180: *APOBR* Thr321_Gly329del9was only detected in the screening sample and not in the independent study groups

The remaining nine variants were located in the un-translated region (UTR) or intronic regions or were synonymous (rs74949322: c.57+50C>T, rs151233: p.Leu22 =, rs149271: p.Glu170 =, rs180744: p.Gln553 =, rs151174: p.Gly560 =, rs40831: p.Ala686 =, rs61738759: p.Pro1012 =, rs40833: c.*218C>G and rs142786317: c.*118_*119delCA; S1 Table). *In silico* analysis showed that the infrequent alleles at these variants caused changes of either transcription factor binding sites splicing enhancer and silencer binding sites, or both (S1 Table).

### SULT1A1

Of the initially detected variants (7 non-synonymous, S2 Data) in the coding region of *SULT1A1* in 95 extremely obese children and adolescents, none could be confirmed with an independent method. A high sequence similarity between the *SULT1A* gene family allowed for unspecific amplification of several *SULT1A* genes for individuals in the mutation screen resulting in artifacts. Hence, apart from *SULT1A1* we also amplified one or more *SULT1* gene family members. So that a variant that seemed to be located in *SULT1A1* was in fact attributable to *SULT1A2*, where it represents the wild type allele (see S2 Data and S2 Table for more detail). For the variant Met1Val, which could not be explained by one of the other *SULT1A* family members, two independent genotyping methods could not replicate our initial uni-directional Sanger-resequencing finding. We hence deemed this variant an artifact.

For the synonymous variants in *SULT1A1*, *in silico* predictions varied. All variants were predicted to change splicing enhancer and silencers, or to directly affect splice sites or transcription factor binding sites (S1 Table). Particularly for the variant p.Pro200 = (rs3176926), both changes in the binding domains of splicing regulators as well for the transcription factor AML-1 were predicted.

### SULT1A2

In *SULT1A2* seven non-synonymous SNPs were detected (rs4149403: p.Ile7Thr, rs10797300: p.Pro19Leu, rs145008170: p.Ser44Asn, rs4987024: p.Tyr62Phe, rs142241142: p.Ala164Val, rs1059491: p.Asn235Thr, rs75191166: p.Lys282Gly); two synonymous SNPs (rs1690407: p.Ser8 =, rs139896537: p.Ala164 =; S1 Table), and five non-coding variants (rs4149406, rs3743963, rs710410, rs762634, rs145790611; S1 Table) were also identified.

For the non-conservative, non-synonymous SNP rs1136703: p.Ile7Thr (c.20T/C), *in silico* analyses did not predict a functional effect (Table 2). For rs10797300: p.Pro19Leu (c.56C/T), *in silico* programs predicted a functional modification (PolyPhen2 and PANTHER, Table 2). The conservative non-synonymous polymorphism rs145008170: p.Ser44Asn (c.131G/A) is located close to Lys48, which is relevant for binding of the xenobiotic p-nitrophenol to the binding pocket of SULT1A2. *In silico* analyses predicted a “non-neutral” (SNAP) or “deleterious” (PANTHER; Table 2) functional change. The infrequent missense variants rs4987024: p.Tyr62Phe (c.185A/T) and rs142241142: p.Ala164Val (c.491C/T) are non-conservative amino acid exchanges which are not located close to the binding pocket of SULT1A2. *In silico*, a higher probability of functional changes was predicted for p.Tyr62Phe than for p.Ala164Val (Poly Phen 2 “probably damaging”), although the analyses revealed mixed results (Table 2). *In silico* prediction mostly interpreted the conservative SNP rs1059491: p.Asn235Thr (c.704A/C) as functionally relevant (“probably damaging” PolyPhen2, “non-neutral” SNAP and “deleterious” SIFT; Table 2). None of the analyzed non-synonymous SNPs in *SULT1A2* showed association with obesity in 355 obesity trios (TDT; Table 3).

*In silico* predictions vary for the two known synonymous SNPs (rs1690407: p.Ser8 =, rs139896537: p.Ala164 =) and the five non-coding variants (rs4149406: c.148+34T/C, rs3743963: c.500−19T/C, rs710410: c.*7T/C, rs762634: c.*14A>G, rs145790611: c.241+39G/A). For each variant, at least a change in either transcription factor binding sites or splicing enhancer or silencer sites was predicted (S1 Table).

### TUFM

In *TUFM*, the previously unknown variant c.3536C>G was detected in the 3’ untranslated region (previously unknown). In addition, non-coding intronic variants were detected (rs7187776: c.-55T>C, rs4788099: c.817+13T>C, rs8061877: c.248−18G>A, and rs61737565: c.922+29C>G). All five variants in *TUFM* are predicted to lead to altered splicing regulator or transcription factor binding sites (S1 Table). For rs8061877, the disruption of a transcription factor binding site was predicted (TFSearch and Consite), although for different transcription factors (SRY, HFH 2, HMG-IY; S1 Table). For the previously unknown variant g.28854194C/G, *in silico* prediction showed a change in splice enhancer binding sites and splicing silencer sites by three programs (S1 Table). Also, alterations in splice sites for this variant were predicted (Mutation Taster) despite the variant being in the non-coding 3’ UTR of *TUFM*.

## Discussion

Previous studies on the causal variation underlying the obesity association of chr16p11.2 mainly focused on the *SH2B1* gene. Mutation screens in humans [14, 24, 25] have revealed a number of mutations that are too infrequent to explain the genome-wide association of the lead SNP with BMI and obesity. The coding variant rs7498665 (*SH2B1*: p.Thr484Ala) was identified in GWAS studies as lead obesity association signal for a linkage disequilibrium block encompassing 1 Mb. However, this SNP showed no functional effect on STAT3 mediated leptin signaling [14] or the phosphorylation of JAK1 or IRS1 in insulin signaling [25]. Hence, variants underlying the genome-wide significant finding may be located outside the *SH2B1* coding region, but in high LD with the original association signal as proposed by Speliotes et al. [2]. We therefore screened the coding region of *APOBR*, *SULT1A1*, *SULT1A2* and *TUFM* for mutations in 95 extremely obese German children and adolescents.

We identified 13 variants in the *APOBR* coding region, three of which were non-synonymous or deletions. These (rs180743: p.Pro428Ala, rs3833080: p.Gly369_Asp370del9, and rs368546180 p.Thr321_Gly329del9) were genotyped in our trios and case-control study groups. The variants p.Pro428Ala and p.Gly369_Asp370del9 are located close to each other; their LD is high (r^2^ = 0.98) and their minor alleles are associated with obesity in our sample (OR = 1.27, 95%CI: 1.09–1.47, p = 0.002; p_Bonferroni corrected_ = 0.026 and p = 0.003; p_Bonferroni corrected_ = 0.039, OR = 1.25, 95% CI: 1.08–1.45, respectively; Table 3) As the LD and proximity of both polymorphisms with the initial lead SNP and the non-synonymous polymorphism rs7498665 (*SH2B1*: p.Thr484Ala) is very high, the obesity association of all variants are dependent signals. Conditional analysis increased all p-values for rs180743, rs3833080, and rs7498665 above 0.7, indicating high signal dependency. Previously association of the minor alleles of both variants with hypercholesterolemia had been described [29]. Of note, the hypercholesterolemia and obesity risk allele C of rs180743 was not associated with weight loss during a 1 year lifestyle intervention in children and adolescents [69] although the position of the SNP is conserved (conservation 66% over 29 species, ENSEMBL). The second deletion (rs368546180: p.Thr321_Gly329del9) was only detected once in our sample of 2,179 obese cases and 435 normal weight or lean controls. Hence, obesity association assessment was not possible. Although variant rs368546180 is in-frame and located in the repeat region of *APOBR* which has no predicted function [27], *in silico* prediction of the variant implicated a potentially reduced function (Table 2).

Almost all initially screened individuals are homozygous carriers of obesity risk alleles at the rs180743 (p.Pro428Ala) and rs3833080 (p.Gly369_Asp370del9) polymorphisms. Our results suggest that an *in vitro* functional validation of both deletion rs3833080 and SNP rs180743 would be of interest. Brown et al [27] suggested a contribution to binding of the specific ligand apoB48 of the repeat region in which both variants are located, with alterations possibly leading to reduced uptake of chylomicrons (CMs) or CM remnants. This hypothesis could be tested by lipoprotein-uptake assays or ligand blotting, as suggested by Daniel et al. [70] and Brown et al. [27].

In *SULT1A2*, none of the variants was associated with obesity (Table 3), so they do not contribute to our initial TDT finding although some of the variants were predicted to have functional effects. Of the detected missense variants, several have known *in vitro* functional effects on xenobiotic sulfonation, e.g. rs4149404 (p.Ile7Thr), rs10797300 (p.Pro19Ser), and rs1059491 (p.Asn235Thr [71]). Glatt et al. [39] reported an obesity association for the minor allele of *SULT1A1* rs141581853 p.Arg213His in *SULT1A1*; the variant was not detected in our screen. In contrast to *SULT1A1* which shows ubiquitous expression [39], the expression of *SULT1A2* is limited to liver, blood platelets, heart, brain and skin. Both sulfotransferases share their substrates [72]. Even if the detected variants entail biological functional changes at the protein level, other sulfotransferases can most likely compensate for the function of each other *in vivo* when only one sulfotransferase is affected [73].

In addition to these variants which directly affect the amino acid composition of the proteins, recent studies detected a cis-regulatory element (intronic SNP rs4788099 in *SH2B1*) which affects the expression of nearby genes (*TUFM*, coiled-coil domain containing 101 gene: *CCDC101*, Homo sapiens spinster homolog 1 gene: *SPNS1*, *SULT1A1* and sulfotransferase family, cytosolic, 1A, phenolpreferring, member 4 gene: *SULT1A4*) in B cells and monocytes [74]. In rodents, differential regulation of the central nervous expression of several genes on chr16p11.2 was shown in reaction to high caloric diets [40, 75]. The GWAS lead SNP rs7359397 also affects the expression of *SULT1A1*, *SPNS1* and *TUFM*, but not *SH2B1* in *cis*. Although the SNP alone only explained 0.0086% of the genetic variance of BMI, the expression changes elicited in the three genes raise this number to 0.5% [76]. These regulatory effects could also contribute to the BMI association signal at chr 16p11.2 in GWAS [2, 77, 78].

## Conclusion

In sum, of the five analyzed genes *SH2B1* and *APOBR* comprised non-synonymous variants associated with obesity. These variants had a medium to high minor allele frequency and were thus previously identified in larger cohorts and population based samples (i.e. 1000genomes and exome variant server). Low frequency variants with potentially major gene effects for weight regulation besides *SH2B1* p.βThr656Ile/γPro674Ser [14] were not detected.

## Supporting Information

S1 Table *In silico* functional prediction of all detected variants in chr16p11.2 (screened genes *APOBR*, *SULT1A1*, and *SULT1A2*). (DOCX)

S2 Table Mutated positions and genomic codons in *SULT1A1* and *SULT1A2*. The genomic codons and the amino acids were taken from the alpha splice variants of *SULT1A1* (ENST00000314752), *SULT1A2* (ENSG00000197165), *SULT1A3* (ENST00000354723), and *SULT1A4* (ENST00000395400). The positions of missense variants which correspond with the amino acid sequence of the other sulfotransferase are marked in grey.
(DOCX)

S3 Table List of primers and screening methods for all analyzed genes (*APOBR, SULT1A1, SULT1A2* and *TUFM*). (DOCX)

S1 Data Location of deletion rs3833080 [*APOBR* p.Gly369_Asp370del9]. (DOCX)

S2 Data Mutation screen of SULT1A1. (DOCX)

S1 Fig DNA sequence of APOBR at the position of Del2 (rs3833080 [Ala345_Gly346delAlaGlyThrAlaSerGlyGlyGluGluAlaGly]). Several potential positions of the deletion are displayed below the wild type sequence of the repeat region. The sequence marked with rs3833080 is given in the SNP database of the National Center for Biotechnology Information (http://www.ncbi.nlm.nih.gov/snp/).
(DOCX)

S2 Fig Multiple sequence alignment of SULT1A1, SULT1A2 SULT1A3 and SULT1A4. The amino acid sequence of the alpha splice variants of SULT1A1 (ENSP00000378971), SULT1A2 (ENSG00000197165), SULT1A3 (ENSP00000346760) and SULT1A4 (ENSP00000378796) were aligned using the program T-Coffee (http://www.ebi.ac.uk/Tools/msa/tcoffee/). Here, an asterisk below the amino acid alignment marks complete sequence identity, while dots below the sequence mark amino acids that share similar side chains or charges. The positions of missense variants detected in the mutation screens are marked in yellow, green marks the variants that could not be verified with independent methods, red marks the variants rs35728980 and rs1059491 which encode Asn235Thr in both SULT1A1 and SULT1A2, respectively.
(DOCX)

S3 Fig Mutated positions in the obesity candidate genes of chr16p11.2 and regional overview. The first picture shows the whole chromosomal region 16p11.2 is displayed with the genes with non-synonymous variants described in this manuscript marked with vertical lines. The underlying picture shows the obesity association signals of SNPs in the GIANT collective (Speliotes et al. 2010). After the whole chromosomal region for reference, the screened genes are depicted with the detected mutations and MAF in CEU according to dbSNP (http://www.ncbi.nlm.nih.gov/projects/SNP/). Here, the horizontal lines symbolize the introns while exons are marked with bars. Functional domains are also included in the graphs. The positions of the variants are indicated with vertical lines.
(DOCX)

## References

[1] Locke AE, Kahali B, Berndt SI, Justice AE, Pers TH, Day FR, et al Genetic studies of body mass index yield new insights for obesity biology. Nature. 2015;518: 197–206. 10.1038/nature14177 25673413 PMC4382211

[2] Speliotes EK, Willer CJ, Berndt SI, Monda KL, Thorleifsson G, Jackson AU, et al Association analyses of 249,796 individuals reveal eighteen new loci associated with body mass index. Nat Genet. 2010;42: 937–948. 10.1038/ng.686 20935630 PMC3014648

[3] Renström F, Payne F, Nordström A, Brito EC, Rolandsson O, Hallmans G, et al Replication and extension of genome-wide association study results for obesity in 4923 adults from northern Sweden. Hum Mol Genet. 2009;18: 1489–1496. 10.1093/hmg/ddp041 19164386 PMC2664142

[4] Shi J, Long J, Gao YT, Lu W, Cai Q, Wen W, et al Evaluation of genetic susceptibility loci for obesity in Chinese women. Am J Epidemiol. 2010;172: 244–254. 10.1093/aje/kwq129 20616199 PMC2917056

[5] Holzapfel C, Grallert H, Huth C, Wahl S, Fischer B, Döring A, et al Genes and lifestyle factors in obesity: results from 12,462 subjects from MONICA/KORA. Int J Obes (Lond). 2010;34: 1538–1545. 10.1038/ijo.2010.79 20386550 PMC3251754

[6] Beckers S, Zegers D, Van Gaal LF, Van Hul W. Replication of the SH2B1 rs7498665 Association with Obesity in a Belgian Study Population. Obes Facts. 2011;4: 473–477. 10.1159/000335305 22248999 PMC6444515

[7] Takeuchi F, Yamamoto K, Katsuya T, Nabika T, Sugiyama T, Fujioka A, et al Association of genetic variants for susceptibility to obesity with type 2 diabetes in Japanese individuals. Diabetologia. 2011;54: 1350–1359. 10.1007/s00125-011-2086-8 21369819

[8] Hester JM, Wing MR, Li J, Palmer ND, Xu J, Hicks PJ, et al Implication of European-derived adiposity loci in African Americans. Int J Obes (Lond). 2012;36: 465–473. 10.1038/ijo.2011.131 21750520 PMC3306054

[9] Elks CE, Loos RJ, Hardy R, Wills AK, Wong A, Wareham NJ, et al Adult obesity susceptibility variants are associated with greater childhood weight gain and a faster tempo of growth: the 1946 British Birth Cohort Study. Am J Clin Nutr. 2012;95: 1150–1156. 10.3945/ajcn.111.027870 22456663 PMC3325838

[10] Robiou-du-Pont S, Bonnefond A, Yengo L, Vaillant E, Lobbens S, Durand E, et al Contribution of 24 obesity-associated genetic variants to insulin resistance, pancreatic beta-cell function and type 2 diabetes risk in the French population. Int J Obes (Lond). 2013;37: 980–985. 10.1038/ijo.2012.175 23090577

[11] Zheng Z, Hong L, Huang X, Yang P, Li J, Ding Y, et al Screening for Coding Variants in FTO and SH2B1 Genes in Chinese Patients with Obesity. PLoS One. 2013;8: e67039 10.1371/journal.pone.0067039 23825611 PMC3692548

[12] Graff M, Ngwa JS, Workalemahu T, Homuth G, Schipf S, Teumer A, et al Genome-wide analysis of BMI in adolescents and young adults reveals additional insight into the effects of genetic loci over the life course. Hum Mol Genet. 2013;22: 3597–3607. 10.1093/hmg/ddt205 23669352 PMC3736869

[13] Poveda A, Ibáñez ME, Rebato E. Common variants in BDNF, FAIM2, FTO, MC4R, NEGR1, and SH2B1 show association with obesity-related variables in Spanish Roma population. Am J Hum Biol. 2014;26: 660–669. 10.1002/ajhb.22576 24948161

[14] Volckmar AL, Bolze F, Jarick I, Knoll N, Scherag A, Reinehr T, et al Mutation screen in the GWAS derived obesity gene SH2B1 including functional analyses of detected variants. BMC Med Genomics. 2012;5: 65 10.1186/1755-8794-5-65 23270367 PMC3544595

[15] Bochukova EG, Huang N, Keogh J, Henning E, Purmann C, Blaszczyk K, et al Large, rare chromosomal deletions associated with severe early-onset obesity. Nature. 2010;463: 666–670. 10.1038/nature08689 19966786 PMC3108883

[16] Bachmann-Gagescu R, Mefford HC, Cowan C, Glew GM, Hing AV, Wallace S, et al Recurrent 200-kb deletions of 16p11.2 that include the SH2B1 gene are associated with developmental delay and obesity. Genet Med. 2010;12: 641–647. 10.1097/GIM.0b013e3181ef4286 20808231

[17] Perrone L, Marzuillo P, Grandone A, del Giudice EM. Chromosome 16p11.2 deletions: another piece in the genetic puzzle of childhood obesity. Ital Jn Pediatr. 2010;36: 43 10.1186/1824-7288-36-43 20540750 PMC2903605

[18] Walters RG, Jacquemont S, Valsesia A, de Smith AJ, Martinet D, Andersson J, et al A new highly penetrant form of obesity due to deletions on chromosome 16p11.2. Nature. 2010;463: 671–675. 10.1038/nature08727 20130649 PMC2880448

[19] Egger JI, Verhoeven WM, Verbeeck W, de Leeuw N. Neuropsychological phenotype of a patient with a de novo 970 kb interstitial deletion in the distal 16p11.2 region. Neuropsychiatr Dis Treat. 2014;10: 513–517. 10.2147/NDT.S58684 24707176 PMC3971941

[20] Jarick I, Vogel CI, Scherag S, Schäfer H, Hebebrand J, Hinney A, et al Novel common copy number variation for early onset extreme obesity on chromosome 11q11 identified by a genome-wide analysis. Hum Mol Genet. 2011;20: 840–852. 10.1093/hmg/ddq518 21131291 PMC3024044

[21] Jacquemont S, Reymond A, Zufferey F, Harewood L, Walters RG, Kutalik Z, et al Mirror extreme BMI phenotypes associated with gene dosage at the chromosome 16p11.2 locus. Nature. 2011;478: 97–102. 10.1038/nature10406 21881559 PMC3637175

[22] Morris DL, Rui L. Recent advances in understanding leptin signalling and leptin resistance. Am J Physiol Endocrinol Metab. 2009;297: E1247–1259. 10.1152/ajpendo.00274.2009 19724019 PMC2793049

[23] Ren D, Zhou Y, Morris D, Li M, Li Z, Rui L. Neuronal SH2B1 is essential for controlling energy and glucose homeostasis. J Clin Invest. 2007;117: 397–406. 17235396 10.1172/JCI29417 PMC1765516

[24] Doche ME, Bochukova EG, Su HW, Pearce LR, Keogh JM, Henning E, et al Human SH2B1 mutations are associated with maladaptive behaviors and obesity. J Clin Invest. 2012;122: 4732–4736. 10.1172/JCI62696 23160192 PMC3533535

[25] Pearce LR, Joe R, Doche ME, Su HW, Keogh JM, Henning E, et al Functional characterization of obesity-associated variants involving the α and β isoforms of human SH2B1. Endocrinology. 2014;155: 3219–3226. 10.1210/en.2014-1264 24971614 PMC4138566

[26] Attia J, Ioannidis JP, Thakkinstian A, McEvoy M, Scott RJ, Minelli C, et al How to use an article about genetic association: A: Background concepts. JAMA. 2009;301: 74–81. 10.1001/jama.2008.901 19126812

[27] Brown ML, Ramprasad MP, Umeda PK, Tanaka A, Kobayashi Y, Watanabe T, et al A macrophage receptor for apolipoprotein B48: cloning, expression, and atherosclerosis. Proc Natl Acad Sci U S A. 2000;97: 7488–7493. 10852956 10.1073/pnas.120184097 PMC16572

[28] Brown ML, Yui K, Smith JD, LeBoeuf RC, Weng W, Umeda PK, et al The murine macrophage apoB-48 receptor gene (Apob-48r): homology to the human receptor. J Lipid Res. 2002;43: 1181–1191. 12177162

[29] Fujita Y, Ezura Y, Bujo H, Nakajima T, Takahashi K, Kamimura K, et al Association of nucleotide variations in the apolipoprotein B48 receptor gene (APOB48R) with hypercholesterolemia. J Hum Genet. 2005;50: 203–209. 15830122 10.1007/s10038-005-0240-1

[30] Lumeng CN, Deyoung SM, Bodzin JL, Saltiel AR. Increased inflammatory properties of adipose tissue macrophages recruited during diet-induced obesity. Diabetes. 2007;56: 16–23. 17192460 10.2337/db06-1076

[31] Haraguchi G, Kobayashi Y, Brown ML, Tanaka A, Isobe M, Gianturco SH, et al PPAR(alpha) and PPAR(gamma) activators suppress the monocyte-macrophage apoB-48 receptor. J Lipid Res. 2003;44: 1224–1231. 12700342 10.1194/jlr.M300077-JLR200

[32] Varela LM, Ortega-Gomez A, Lopez S, Abia R, Muriana FJ, Bermudez B. The effects of dietary fatty acids on the postprandial triglyceride-rich lipoprotein/apoB48 receptor axis in human monocyte/macrophage cells. J Nutr Biochem. 2013;24: 2031–2039. 10.1016/j.jnutbio.2013.07.004 24231096

[33] Varela LM, Ortega A, Bermudez B, Lopez S, Pacheco YM, Villar J, et al A high-fat meal promotes lipid-load and apolipoprotein B-48 receptor transcriptional activity in circulating monocytes. Am J Clin Nutr. 2011;93: 918–925. 10.3945/ajcn.110.007765 21367954

[34] Harris RM, Waring RH, Kirk CJ, Hughes PJ. Sulfation of "estrogenic" alkylphenols and 17beta-estradiol by human platelet phenol sulfotransferases. J Biol Chem. 2000;275: 159–166. 10617600 10.1074/jbc.275.1.159

[35] Ghose R, Omoluabi O, Gandhi A, Shah P, Strohacker K, Carpenter KC, et al Role of high-fat diet in regulation of gene expression of drug metabolizing enzymes and transporters. Life Sci. 2011;89: 57–64. 10.1016/j.lfs.2011.05.005 21620874 PMC3156825

[36] Mahabir S, Baer DJ, Giffen C, Clevidence BA, Campbell WS, Taylor PR, et al Comparison of energy expenditure estimates from 4 physical activity questionnaires with doubly labeled water estimates in postmenopausal women. Am J Clin Nutr. 2006;84: 230–236. 16825700 10.1093/ajcn/84.1.230

[37] Emaus A, Espetvedt S, Veierød MB, Ballard-Barbash R, Furberg AS, Ellison PT, et al 17-beta-estradiol in relation to age at menarche and adult obesity in premenopausal women. Hum Reprod. 2008;23: 919–927. 10.1093/humrep/dem432 18227106

[38] Rodríguez-Moran M, Guerrero-Romero F, Aradillas-García C, Violante R, Simental-Mendia LE, Monreal-Escalante E, et al Obesity and family history of diabetes as risk factors of impaired fasting glucose: implications for the early detection of prediabetes. Pediatr Diabetes. 2010;11: 331–336. 10.1111/j.1399-5448.2009.00590.x 19895410

[39] Glatt H, Boeing H, Engelke CE, Ma L, Kuhlow A, Pabel U, et al Human cytosolic sulphotransferases: genetics, characteristics, toxicological aspects. Mutat Res. 2001;482: 27–40. 11535246 10.1016/s0027-5107(01)00207-x

[40] Gutierrez-Aguilar R, Kim DH, Woods SC, Seeley RJ. Expression of new loci associated with obesity in diet-induced obese rats: from genetics to physiology. Obesity (Silver Spring). 2012;2: 306–312. 10.1038/oby.2011.236 21779089

[41] Ling M, Merante F, Chen HS, Duff C, Duncan AM, Robinson BH. The human mitochondrial elongation factor tu (EF-Tu) gene: cDNA sequence, genomic localization, genomic structure, and identification of a pseudogene. Gene. 1997;197: 325–336. 9332382 10.1016/s0378-1119(97)00279-5

[42] Knoll N, Jarick I, Volckmar AL, Klingenspor M, Illig T, Grallert H, et al Gene set of nuclear-encoded mitochondrial regulators is enriched for common inherited variation in obesity. PLoS One. 2013;8: e55884 10.1371/journal.pone.0055884 23409076 PMC3568071

[43] Santos AR, Duarte CB. Validation of internal control genes for expression studies: effects of the neurotrophin BDNF on hippocampal neurons. J Neurosci Res. 2008;86: 3684–3692. 10.1002/jnr.21796 18655199

[44] Hinney A, Volckmar AL, Antel J. Genes and the hypothalamic control of metabolism in humans. Best Pract Res Clin Endocrinol Metab. 2014;28: 635–647. 10.1016/j.beem.2014.04.007 25256760

[45] Scherag A, Jarick I, Grothe J, Biebermann H, Scherag S, Volckmar AL, et al Investigation of a genome wide association signal for obesity: synthetic association and haplotype analyses at the melanocortin 4 receptor gene locus. PLoS One. 2010;5: e13967 10.1371/journal.pone.0013967 21085626 PMC2981522

[46] Hinney A, Nguyen TT, Scherag A, Friedel S, Brönner G, Müller TD, et al Genome wide association (GWA) study for early onset extreme obesity supports the role of fat mass and obesity associated gene (FTO) variants. PloS One. 2007;2: e1361 18159244 10.1371/journal.pone.0001361 PMC2137937

[47] Scherag A, Dina C, Hinney A, Vatin V, Scherag S, Vogel CI, et al Two new Loci for body-weight regulation identified in a joint analysis of genome-wide association studies for early-onset extreme obesity in French and German study groups. PloS Genet. 2010;6: e1000916 10.1371/journal.pgen.1000916 20421936 PMC2858696

[48] Hinney A, Hohmann S, Geller F, Vogel C, Hess C, Wermter AK, et al Melanocortin-4 receptor gene: case-control study and transmission disequilibrium test confirm that functionally relevant mutations are compatible with a major gene effect for extreme obesity. J Clin Endocrinol Metab. 2003;88: 4258–4267. 12970296 10.1210/jc.2003-030233

[49] Hayashi K, Yandell DW. How sensitive is PCR-SSCP? Hum Mutat. 1993;2: 338–346. 8257985 10.1002/humu.1380020503

[50] Liu J, Stewart JT. Quantitation of trimipramine enantiomers in human serum by enantioselective high-performance liquid chromatography and mixed-mode disc solid-phase extraction. J Chromatogr B Biomed Sci Appl. 1997;700: 175–182. 9390727 10.1016/s0378-4347(97)00324-1

[51] O'Donovan MC, Oefner PJ, Roberts SC, Austin J, Hoogendoorn B, Guy C, et al Blind analysis of denaturing high-performance liquid chromatography as a tool for mutation detection. Genomics. 1998;52: 44–49. 9740670 10.1006/geno.1998.5411

[52] Jones AC, Austin J, Hansen N, Hoogendoorn B, Oefner PJ, Cheadle JP, et al Optimal temperature selection for mutation detection by denaturing HPLC and comparison to single-stranded conformation polymorphism and heteroduplex analysis. Clin Chem. 1999;45: 1133–1140. 10430776

[53] Kurelac I, Lang M, Zuntini R, Calabrese C, Simone D, Vicario S, et al Searching for a needle in the haystack: comparing six methods to evaluate heteroplasmy in difficult sequence context. Biotechnol Adv. 2012;30: 363–371. 10.1016/j.biotechadv.2011.06.001 21689740

[54] Reinehr T, Hinney A, de Sousa G, Austrup F, Hebebrand J, Andler W. Definable somatic disorders in overweight children and adolescents. J Pediatr. 2007;150: 618–622. 17517246 10.1016/j.jpeds.2007.01.042

[55] Purcell S, Neale B, Todd-Brown K, Thomas L, Ferreira MA, Bender D, et al PLINK: a tool set for whole-genome association and population-based linkage analyses. Am J Hum Genet. 2007;81: 559–575. 17701901 10.1086/519795 PMC1950838

[56] Spielman RS, McGinnis RE, Ewens WJ. Transmission test for linkage disequilibrium: the insulin gene region and insulin-dependent diabetes mellitus (IDDM). Am J Hum Genet. 1993;52: 506–516. 8447318 PMC1682161

[57] R Core Team. R: A language and environment for statistical computing. R Foundation for Statistical Computing, Vienna, Austria. 2014. URL http://www.R-project.org/.

[58] Cartegni L, Wang J, Zhu Z, Zhang MQ, Krainer AR. ESEfinder: a web resource to identify exonic splicing enhancers. Nucleic Acid Res. 2003;31: 3568–3571. 12824367 10.1093/nar/gkg616 PMC169022

[59] Goren A, Ram O, Amit M, Keren H, Lev-Maor G, Vig I, et al Comparative analysis identifies exonic splicing regulatory sequences—The complex definition of enhancers and silencers. Mol Cell. 2006;22: 769–781. 16793546 10.1016/j.molcel.2006.05.008

[60] Fairbrother WG, Yeh RF, Sharp PA, Burge CB. Predictive identification of exonic splicing enhancers in human genes. Science. 2002;297: 1007–1013. 12114529 10.1126/science.1073774

[61] Heinemeyer T, Wingender E, Reuter I, Hermjakob H, Kel AE, Kel OV, et al Databases on Transcriptional Regulation: TRANSFAC, TRRD, and COMPEL. Nucleic Acids Res. 1998;26: 364–370. 10.1093/nar/26.1.362 PMC147251 9399875

[62] Sandelin A, Wasserman WW, Lenhard B. ConSite: web-based prediction of regulatory elements using cross-species comparison. Nucleic Acids Res. 2004;32: W249–252. 15215389 10.1093/nar/gkh372 PMC441510

[63] Mi H, Muruganujan A, Thomas PD. PANTHER in 2013: modeling the evolution of gene function, and other gene attributes, in the context of phylogenetic trees. Nucl. Acids Res. 2013;41: D377–386. 10.1093/nar/gks1118 23193289 PMC3531194

[64] Ramensky V, Bork P, Sunyaev S. Human non-synonymous SNPs: server and survey. Nucleic Acids Res. 2002;30: 3894–3900. 12202775 10.1093/nar/gkf493 PMC137415

[65] Bromberg Y, Rost B. SNAP: predict effect of non-synonymous polymorphisms on function. Nucleic Acids Res. 2007;35: 3823–3835. 17526529 10.1093/nar/gkm238 PMC1920242

[66] Ferrer-Costa C, Orozco M, de la Cruz X. Sequence-based prediction of pathological mutations. Proteins. 2004;57: 811–819. 15390262 10.1002/prot.20252

[67] Schwarz JM, Rödelsperger C, Schuelke M, Seelow D. MutationTaster evaluates disease-causing potential of sequence alterations. Nat Methods. 2010;7: 575–576. 10.1038/nmeth0810-575 20676075

[68] Choi Y, Sims GE, Murphy S, Miller JR, Chan AP. Predicting the Functional Effect of Amino Acid Substitutions and Indels. PLoS ONE. 2012;7: e46688 10.1371/journal.pone.0046688 23056405 PMC3466303

[69] Volckmar AL, Pütter C, Song JY, Graninger J, Knoll N, Wolters B, et al Analyses of Non-Synonymous Obesity Risk Alleles in SH2B1 (rs7498665) and APOB48R (rs180743) in Obese Children and Adolescents Undergoing a 1-year Lifestyle Intervention. Exp Clin Endocrinol Diabetes. 2013;121: 1–4. 10.1055/s-0031-1297992 23519644

[70] Daniel TO, Schneider WJ, Goldstein JL, Brown MS. Visualization of lipoprotein receptors by ligand blotting. J Biol Chem. 1983;258: 4606–4611. 6300091

[71] Meinl W, Meerman JH, Glatt H. Differential activation of promutagens by alloenzymes of human sulfotransferase 1A2 expressed in Salmonella typhimurium. Pharmacogenetics. 2002;12: 677–689. 12464797 10.1097/00008571-200212000-00002

[72] Glatt H, Seidel A, Harvey RG, Coughtrie MW. Activation of benzylic alcohols to mutagens by human hepatic sulphotransferases. Mutagenesis. 1994;9: 553–557. 7854148 10.1093/mutage/9.6.553

[73] Glatt H, Meinl W. Pharmacogenetics of soluble sulfotransferases (SULTs). Naunyn Schmiedebergs Arch Pharmacol. 2004;369: 55–68. 14600802 10.1007/s00210-003-0826-0

[74] Guo Y, Lanktree MB, Taylor KC, Hakonarson H, Lange LA, Keating BJ, et al Gene-centric meta-analyses of 108 912 individuals confirm known body mass index loci and reveal three novel signals. Hum Mol Genet. 2013;22: 184–201. 10.1093/hmg/dds396 23001569 PMC3522401

[75] Yoganathan P, Karunakaran S, Ho MM, Clee SM. Nutritional regulation of genome-wide association obesity genes in a tissue-dependent manner. Nutr Metab (Lond). 2012;1: 65 10.1186/1743-7075-9-65 22781276 PMC3537611

[76] Huan T, Liu C, Joehanes R, Zhang X, Chen BH, Johnson AD, et al A systematic heritability analysis of the human whole blood transcriptome. Hum Genet. 2015;134: 343–358. 10.1007/s00439-014-1524-3 25585846 PMC4339826

[77] Willer CJ, Speliotes EK, Loos RJ, Li S, Lindgren CM, Heid IM, et al Six new loci associated with body mass index highlight a neuronal influence on body weight regulation. Nat Genet. 2009;41: 25–34. 10.1038/ng.287 19079261 PMC2695662

[78] Thorleifsson G, Walters GB, Gudbjartsson DF, Steinthorsdottir V, Sulem P, Helgadottir A, et al Genome-wide association yields new sequence variants at seven loci that associate with measures of obesity. Nat Genet. 2009;41: 18–24. 10.1038/ng.274 19079260

