# Supplementary material for: Fine Mapping of a GWAS-Derived Obesity Candidate Region on Chromosome 16p11.2
Source: PLoS One. 2015 May 8;10(5):e0125660. doi: 10.1371/journal.pone.0125660 (PMC4425372; doi:10.1371/journal.pone.0125660)
Supplement: S1 Table — (DOCX) [file pone.0125660.s001.docx]

**Supplementary Table 1:** ***In silico* functional prediction of all detected variants in chr16p11.2 (screened genes *APOBR, SULT1A1*, and *SULT1A2)***

|  |  |  |  | **ESEfinder** | **ESRSearch** | **RESCUE_ESE** | **TFSearch** ^a^ | **Consite** ^a^ | **Mutation Taster** | |  |
| --- | --- | --- | --- | --- | --- | --- | --- | --- | --- | --- | --- |
| **Gene** | **rs-Number** | **Amino acid changes** | **DNA position** | *Splice sites* | *Splice sites* | *Splice sites* | *transcription factor binding sites* | *transcription factor binding sites* | *Prediction* | *Prob.* | |
| ***APOBR*** | rs74949322 | - | c.57+50C>T | changed | changed | not changed | not changed | changed | Polymorphism | 0.73 | |
|  | rs151233 | Leu22= | c.75C/T | not changed | not changed | not changed | not changed | not changed | Polymorphism | 0.71 | |
|  | rs149271 | Glu170= | c.520A/G | changed | changed | changed | not changed | not changed | Polymorphism | 0.71 | |
|  | rs368546180 | Thr321_Gly329del9 | c.933_934delA  CAGCCTCAG  GCGGGGAGG  AGGCTGAA | NA | NA | NA | changed | changed | Polymorphism | 0.71 | |
|  | rs3833080 | Gly369_Asp370del9 | c.1035_1036del  GGGACAGCC  TCAGGAGGG  GAGGAGGCC | NA | NA | NA | changed | changed | Polymorphism | 1 | |
|  | rs180743 | Pro428Ala | c.1282C>G | changed | not changed | not changed | changed | changed | Polymorphism | 1 | |
|  | rs180744 | Gln562= | c.1686A>G | changed | changed | changed | not changed | not changed | Polymorphism | 0.71 | |
|  | rs151174 | Gly569= | c.1707C>T | changed | changed | changed | not changed | possible | Polymorphism | 0.72 | |
|  | rs40831 | Ala695= | c.2085A>G | changed | not changed | not changed | changed | changed | Polymorphism | 0.71 | |
|  | rs200751685 | Asp994= | c.2982C>T | changed | changed | changed | not changed | possible | Polymorphism | 0.71 | |
|  | rs61738759 | Pro1021= | c.3063G>A | changed | changed | changed | not changed | not changed | Polymorphism | 0.72 | |
|  | rs40833 | - | c.*218C>G | changed | not changed | changed | changed | changed | Polymorphism | 0.73 | |
|  | rs142786317 | - | c.*118_*119insA | changed | changed | changed | changed | changed | Polymorphism | 0.71 | |

|  |  |  |  | **ESEfinder** | **ESRSearch** | **RESCUE_ESE** | **TFSearch** ^a^ | **Consite** ^a^ | **Mutation Taster** | |
| --- | --- | --- | --- | --- | --- | --- | --- | --- | --- | --- |
| **Gene** | **rs-Number** | **Amino acid changes** | **DNA position** | *Splice sites* | *Splice sites* | *Splice sites* | *transcription factor binding sites* | *transcription factor binding sites* | *Prediction* | *Prob.* |
| ***SULT1A1*** | rs34513973 | Pro19= | c.57G>A | not changed | not changed | not changed | not changed | not changed | Disease causing | 0.99 |
|  | rs1126446 | Thr51= | c.153T>C | not changed | changed | changed | not changed | Snail | Polymorphism | 0.98 |
|  | rs1126447 | Val54= | c.162A>G | changed | not changed | not changed | p300 | Thing1-E47, c-REL | Polymorphism | 1.77 |
|  | rs1042005 | Lys147= | c.441G>A | changed | not changed | not changed | not changed | Snail | Polymorphism | 0.99 |
| ***SULT1A1*** | rs3176926 | Pro200= | c.600G>C | changed | changed | changed | AML-1a | AML-1 | Polymorphism | 2.99 |
|  | rs35497673 | Thr219= | c.657C>T | not changed | not changed | not changed | not changed | not changed | Polymorphism | 0.99 |
| ***SULT1A2*** | rs710410 | NA | c.*7T/C | changed | changed | not changed | not changed | not changed | Polymorphism | 0.71 |
|  | rs1136703 | Ile7Thr | c.20T/C | changed | not changed | not changed | not changed | not changed | Polymorphism | 1 |
|  | rs1690407 | Ser8= | c.24T/C | changed | not changed | not changed | not changed | p50 | Polymorphism | 0.72 |
|  | rs10797300 | Pro19Leu | c.56C/T | not changed | changed | changed | GATA1 | Snail | Polymorphism | 0.99 |
|  | rs4149406 | NA | c.148+34T/C | changed | not changed | not changed | not changed | not changed | Polymorphism | 0.72 |
|  | rs145008170 | Ser44Asn | c.131G/A | not changed | changed | not changed | deltaE | cFOS | Polymorphism | 0.97 |
|  | NA | NA | c.241+39G/A | changed | not changed | not changed | not changed | not changed | Polymorphism | 0.71 |
|  | rs4987024 | Tyr62Phe | c.185A/T | changed | changed | not changed | Nkx-2 | Snail | Polymorphism | 0.99 |
|  | rs3743963 | NA | c.500-19T/C | not changed | not changed | not changed | not changed | RXR-VDR | Polymorphism | 0.73 |
|  | rs142241142 | Ala164Val | c.491C/T | changed | changed | changed | not changed | not changed | Polymorphism | 1 |
|  | rs139896537 | Ala164= | c.492T/C | changed | not changed | not changed | not changed | E74A | Polymorphism | 0.73 |
|  | rs1059491 | Asn235Thr | c.704A/C | not changed | changed | not changed | not changed | CFI-USP | Polymorphism | 0.04 |
|  | rs762634 | NA | c.*14A>G | changed | not changed | not changed | not changed | not changed | Polymorphism | 0.73 |

|  |  |  |  | **ESEfinder** | **ESRSearch** | **RESCUE_ESE** | **TFSearch** ^a^ | | **Consite** ^a^ | | **Mutation Taster** | | |
| --- | --- | --- | --- | --- | --- | --- | --- | --- | --- | --- | --- | --- | --- |
| **Gene** | **rs-Number** | **Amino acid changes** | **DNA position** | *Splice sites* | *Splice sites* | *Splice sites* | | *transcription factor binding sites* | | *transcription factor binding sites* | *Prediction* | *Prob.* | |
| ***TUFM*** | rs7187776 | NA | c.-55T>C | changed | changed | not changed | | not changed | | not changed | Polymorphism | 0.85 |  |
|  | rs8061877 | NA | c.248-18G>A | not changed | changed | not changed | | SRY | | HFH 2, HMG-IY | Polymorphism | 0.99 |  |
|  | rs61737565 | NA | c.922+29C>G | changed | changed | not changed | | not changed | | SU h | Polymorphism | 0.82 |  |
|  | rs4788099 | NA | c.817+13T>C | not changed | changed | not changed | | not changed | | Thing1-E47 | Polymorphism | 0.71 |  |
|  | NA | NA | g.28854194C>G | changed | changed | changed | | not changed | | Hen1 | Polymorphism | 0.99 |  |

NA: Not available
