# Supplementary material for: Fine Mapping of a GWAS-Derived Obesity Candidate Region on Chromosome 16p11.2
Source: PLoS One. 2015 May 8;10(5):e0125660. doi: 10.1371/journal.pone.0125660 (PMC4425372; doi:10.1371/journal.pone.0125660)
Supplement: S2 Table — The genomic codons and the amino acids were taken from the alpha splice variants of SULT1A1 (ENST00000314752), SULT1A2 (ENSG00000197165), SULT1A3 (ENST00000354723), and SULT1A4 (ENST00000395400). The positions of missense variants which correspond with the amino acid sequence of the other sulfotransferase are marked in grey. (DOCX) [file pone.0125660.s002.docx]

**Supplementary Table 2: Mutated positions and genomic codons in *SULT1A1* and *SULT1A2***

The genomic codons and the amino acids were taken from the alpha splice variants of *SULT1A1* (ENST00000314752), *SULT1A2* (ENSG00000197165), *SULT1A3* (ENST00000354723), and *SULT1A4* (ENST00000395400). The positions of missense variants which correspond with the amino acid sequence of the other sulfotransferase are marked in grey.

| **rs number** | **AA**  **position** | ***SULT1A1*** | | ***SULT1A2*** | | **SULT1A3** | | **SULT1A4** | | **Confirmed by independent method** |
| --- | --- | --- | --- | --- | --- | --- | --- | --- | --- | --- |
|  |  | **codon** | **amino acid** | **codon** | **amino acid** | **codon** | **amino acid** | **codon** | **amino acid** |  |
|  | 1 | ATG>CTG | Met>Leu | ATG | Met | ATG | Met | ATG | Met | no |
| **rs4149404** | 7 | ACC | Thr | ATC>ACC | Ile>Thr | ACC | Thr | ACC | Thr | yes |
| **rs10797300** | 19 | CCG | Pro | CCG>CTG | Pro>Leu | CCG | Pro | CCG | Pro | yes |
| **rs145008170** | 44 | AGC | Ser | AGC>AAC | Ser>Asn | AAC | Asn | AAC | Asn | yes |
| **rs4987024** | 62 | TAC | Tyr | TAC>AAC | Tyr>Phe | TAC | Tyr | TAC | Tyr | yes |
| **rs1042011** | 151 | GAG>CAG# | Glu>Gln | CAC | His | GAG | Glu | GAG | Glu | no |
| **rs1042014** | 151 | GAG>GAC# | Glu>Asp | CAC | His | GAG | Glu | GAG | Glu | no |
| **rs142241142** | 164 | GTT | Val | GCT>GTT | Ala>Val | GCT | Ala | GCT | Ala | yes |
|  | 222 | TTC>CTC | Phe>Leu | CTC | Leu | TTC | Phe | TTC | Phe | no |
| **rs1801030** | 223 | GTG>ATG | Val>Met | ATG | Met | ATG | Met | ATG | Met | no |
| **rs35728980, rs1059491** | 235 | AAC>ACC | Asn>Thr | AAC>ACC | Asn>Thr | AAC | Asn | AAC | Asn | no* |
|  | 244 | CCC>CGC | Pro>Arg | CGC | Arg | CCC | Pro | CCC | Pro | no |
|  | 245 | CAG>CGG | Gln>Arg | CGG | Arg | CAG | Gln | CAG | Gln | no |

#both SNPs rs1042011 and rs1042014 taken together change the triplet from GAG (Glu) to CAC (His).

*the assay (MALDI TOF) probably detected the same position in both genes due to the high genetic similarity between them, hence the genotypes for both SNPs (rs35728980, rs1059491) have high mendelian errors and were excluded from the analysis.
