# Supplementary material for: Fine Mapping of a GWAS-Derived Obesity Candidate Region on Chromosome 16p11.2
Source: PLoS One. 2015 May 8;10(5):e0125660. doi: 10.1371/journal.pone.0125660 (PMC4425372; doi:10.1371/journal.pone.0125660)
Supplement: S3 Table — (DOCX) [file pone.0125660.s003.docx]

**Supplementary Table 3: List of primers and screening methods for all analyzed genes (*APOBR, SULT1A1*, *SULT1A2* and *TUFM*)**

| **Gene** | **Fragment** | **Primer forward** | **Primer reverse** | **Fragment**  **length [bp]** | **Method** |
| --- | --- | --- | --- | --- | --- |
| ***APOBR*** | F1 | 5`- AAG GTT GGC TTG TCT TGG TG -3' | 3`- ATC AGA AGG AGG AGG GGA AA -5' | 231 | SSCP |
|  | F2 | 5`- AAA GGG CTG GGA CTC TCC T -3' | 3`- GCA GCC TTG GCT GTC TCC -5' | 377 | dHPLC |
|  | F3 | 5`- TGG GGA GCT CAG CTG TAG AA -3' | 3`- CAG GCC TTC TCC ACC ACT AC -5' | 496 | dHPLC |
|  | F4 | 5`- AGA TGG AGC AGG GGG TCA G -3' | 3`- CTC CTC TTT GCC TGA GGT TG -5' | 473 | dHPLC |
|  | F5 | 5`- GGA AGC CAG GAC AAT CTC A -3' | 3`- CAG GTC TAC CTG GCC CTC A -5' | 499 | dHPLC |
|  | F6 | 5`- GTC CTG GGC ACT GAA AGA AC -3' | 3`- TTT GGT GAC GCT GTG TGT G -5' | 393 | dHPLC |
|  | F7 | 5`- TCA GAT GGA GAG GCT GAA GG -3' | 3`- CCG CAT CCT CCT GAG TAT TT -5' | 397 | dHPLC |
|  | F8 | 5`- CGG GGT CTG TAA AGC CTG A -3' | 3`- GCA TAG GCC TCT CCC AGT G -5' | 387 | dHPLC |
|  | F9 | 5`- TGT GGA ACT GAG GAG GGA GA -3' | 3`- AGC CCT CCA GAC CAA ATT CT -5' | 379 | dHPLC |
|  | F10 | 5`- GCT GGT GAA GCT TTG GAA GG -3' | 3`- CCC TCA CTG TCT TCC CTC TG -5' | 374 | dHPLC |
|  | F11 | 5`- ATG GGA GCC ATG GTG GAG -3' | 3`- TCG GGA TTC CAC TCG TTC -5' | 400 | dHPLC |
|  | F12 | 5`- AAC GAG TGG AAT CCC GAA G -3' | 3`- CTG AGT TGC CCT GTG CCT AC -5' | 344 | dHPLC |
|  | F13 | 5`- GTA GGC ACA GGG CAA CTC AG -3' | 3`- GAG GTG GTC TCA GTG GGG TA -5' | 394 | dHPLC |
|  | F14 | 5`- TAC CCC ACT GAG ACC ACC TC -3' | 3`- TCC CTC AGT CTT CCT TGC AG -5' | 240 | SSCP |
| ***SULT1A1*** | F1 | 5`- TTC CAC GCC AAC TTC AAC TA -3' | 3`- CTT GAT CCC CAA GTC CCT G -5' | 213 | SSCP |
|  | F2 | 5`- ACT TTG CAT TTT GGA ATG GT -3' | 3`- ACA CAC ACA AAA AGA TAC TGA TAA CAT -5' | 209 | SSCP |
|  | F3 | 5`- CTG AGT GGC TTT GTG AGT GC -3' | 3`- GAG ATG GGA GGT GAG CAG G -5' | 378 | dHPLC |
|  | F4 | 5`- CCT CAG CCT GCT CAC CTC -3' | 3`- GTG CTC TCA AAC TCC AAC C -5' | 443 | dHPLC |
|  | F5 | 5`- CGT GCC TTG CTC CAG ATT G -3' | 3`- ACC ACC CCT TAG CTC CAC A -5' | 426 | dHPLC |
|  | F6 | 5`- GGA GAA GTT CAT GGT CGG AG -3' | 3`- CAG GAG TCA CAT GGA GGG AA -5' | 252 | dHPLC |
|  | F7 | 5`- AGT ATC CGA GCC TCC ACT G -3' | 3`- AAA GCT GGA GTC TCA TCC CCA -5' | 320 | dHPLC |
|  | F8 | 5`- TGG GAT GAG ACT CCA GCT TT-3' | 3`- CCT GTC CTC CAG TGA TCC TC -5' | 479 | dHPLC |
| ***SULT1A2*** | F1 | 5' - GTG ATG GTG GTA AGG GAA CG -3' | 3'- GTG AGC AGG CTG AGG TGA G -5' | 281 | SSCP |
| **Gene** | **Fragment** | **Primer forward** | **Primer reverse** | **Fragment**  **length [bp]** | **Method** |
| ***SULT1A2*** | F2 | 5'- CTC ACC TCA GCC TGC TCA C -3' | 3'- CAC CTC AGC CTC CCA AAG TA -5' | 409 | dHPLC |
|  | F3 | 5'- AAA CCA AGA GAT GAG CTG GC -3' | 3'- AGG TCC CTG TGA AGT GCC T -5' | 453 | dHPLC |
|  | F4 | 5'- AGC AAT CCA AGC CTC CAC T -3' | 3'- AAA GCT GGA GTC TCA TCC CA -5' | 319 | dHPLC |
|  | F5 | 5'- GGA TGA GAC TCC AGC TTT GC -3' | 3'- CAG CTC AGG GTT TCT CTT GG -5' | 278 | SSCP |
| ***TUFM*** | F1 | 5'- CTC CAT CAT ACT CCG CCC T -3' | 3'- ACC TAC CAC TCC CCC AAA GT -5' | 329 | dHPLC |
|  | F2 | 5'- ACT TCA GCG GTG AGT GGT CA -3' | 3'- AGA CAC TCT GCT GGC CTT G -5' | 396 | dHPLC |
|  | F3 | 5'- AGC TCT GCC TCT AGC ACT GG -3' | 3'- TCC CCA CAA GCC TAA CAT TT -5' | 298 | dHPLC |
|  | F4 | 5'- AGC TCG TTG AAC TTG GCT GT -3' | 3'- CAC TTC CCA AGA CAC AAA GCA -5' | 484 | dHPLC |
|  | F5 | 5'- TGC TTT GTG TCT GGG AAG TG -3' | 3'- GAG ACA GAG GGA AGG CAC AA -5' | 487 | dHPLC |
|  | F6 | 5'- GTG CCT TCC CTC TGT CTC AC -3' | 3'- CCC TCC ACC CTA CAT TCC T -5' | 462 | dHPLC |
|  | F7 | 5'- TAA GGA ATG AAG GCA CCC TG -3' | 3'- ACC CTT TTT GTC CTC CCC TA -5' | 473 | dHPLC |
