# Supplementary material for: Fine Mapping of a GWAS-Derived Obesity Candidate Region on Chromosome 16p11.2
Source: PLoS One. 2015 May 8;10(5):e0125660. doi: 10.1371/journal.pone.0125660 (PMC4425372; doi:10.1371/journal.pone.0125660)
Supplement: S1 Data — (DOCX) [file pone.0125660.s004.docx]

**Location of deletion rs3833080 [*APOBR* p.Gly369_Asp370del9]**

In the repeat region of *APOBR*, an amino acid motif [GluGluAlaGlyThrAlaSerGlyGlyr] is repeated three times exactly and 8 times with small alterations [26]. This repeat region includes a small intron which correlates with one exact repeat length of 27 bps. The deletion we detected covers one of the exact repeats and could therefore either affect the coding sequence of the repeat region (e.g. reducing the total number of amino acid repeats) or cut out the small intron which would leave the coding sequence unaffected. The dbSNP lists an InDel (rs3833080) of the same 27bp sequence with a coding outcome and a comparable minor allele frequency (MAF CEU 0.45). We therefore assume that the detected deletion is the coding deletion rs3833080 [*APOBR* p.Gly369_Asp370del9] (Figure S1).


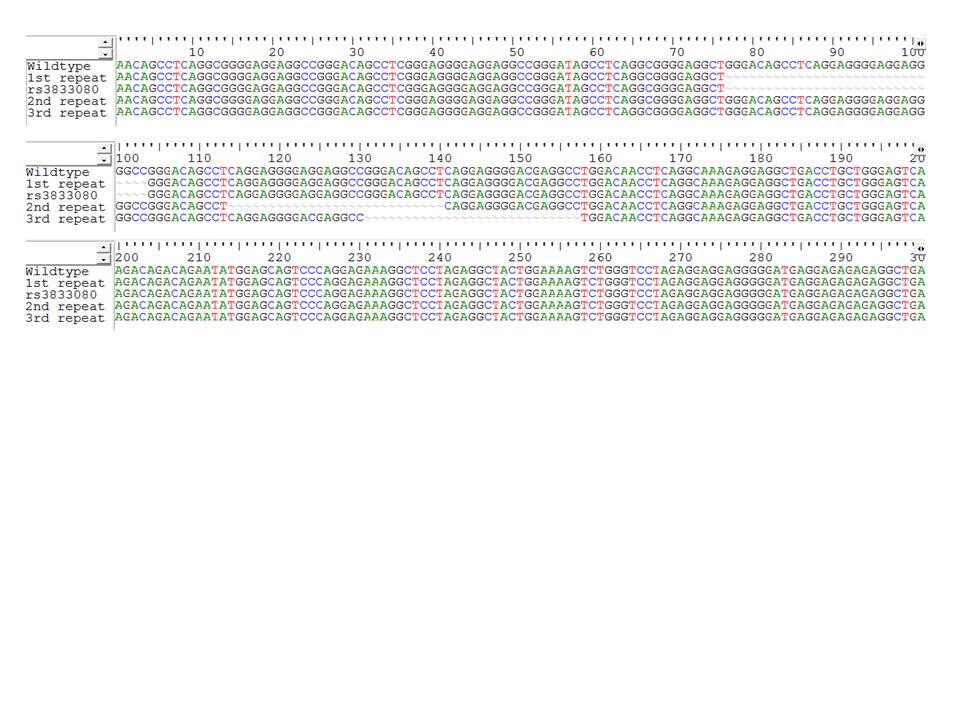


**Supplementary Figure 1:** DNA sequence of APOBR at the position of Del2 (rs3833080 [Ala345_Gly346delAlaGlyThrAlaSerGlyGlyGluGluAlaGly]). Several potential positions of the deletion are displayed below the wild type sequence of the repeat region. The sequence marked with rs3833080 is given in the SNP database of the National Center for Biotechnology Information (http://www.ncbi.nlm.nih.gov/snp/).
