# Supplementary material for: Fine Mapping of a GWAS-Derived Obesity Candidate Region on Chromosome 16p11.2
Source: PLoS One. 2015 May 8;10(5):e0125660. doi: 10.1371/journal.pone.0125660 (PMC4425372; doi:10.1371/journal.pone.0125660)
Supplement: S2 Data — (DOCX) [file pone.0125660.s005.docx]

**Mutation screen of *SULT1A1***

The mutation screen of the coding region of sulfotransferase 1A1 (*SULT1A1*) in 95 extremely obese children and adolescents revealed a total of 15 variants, seven of these non-synonymous (g.390A/C: Met1Leu, rs1042011: Glu151Gln, rs1042014: Glu151Asp, g.17420T/C: Phe222Leu, rs1801030: Val223Met, rs35728980: Asn235Thr, g.17487C/G: Pro244Arg, g.17490 A/G: Gln245Arg).

The *SULT1A* family (SULT1A1 [NM ID: 6817], *SULT1A2* [NM ID: 6799], *SULT1A3* [NM ID: 6818], *SULT1A4* [NM ID: 445329]) of genes displays a high homology (see also Justenhoven 2012) which likely led to an additional amplification of at least SULT1A2 in some cases. We identified aberrant patterns in the mutation screen via SSCP and dHPLC and Sanger re-sequenced individuals with such pattern. We identified seven variants that we initially attributed to *SULT1A1* (*SULT1A1*: g.390A/C: Met1Leu, rs1042011: Glu151Gln, rs1801030: Val223Met, g.17429C/G: Gln225Glu, rs35728980: Asn235Thr, g.17487C/G: Pro244Arg, g.17490 A/G: Gln245Arg). Comparison of the nucleotide sequence of *SULT1A1* and *SULT1A2* reveals that the deviant positions detected in *SULT1A1* for Glu151Gln, Glu151Asp, Phe222Leu, Gln223Glu, Pro244Arg, and Gln245Arg are the amino acids at the corresponding position in *SULT1A2* (see Figure S2 and Table S2). Despite the primer design being chosen for uniqueness in the human genome (http://genome.ucsc.edu/), the amplification of other unwanted fragments can never be fully excluded. As not all individuals were affected (e.g. carrying mutations), most likely the presence of further SNPs in the primer binding sites of the other SULT1A members allowed for this unspecific amplification.

For *SULT1A1* Met1Leu, Sanger re-sequencing revealed this variant allele in one direction only; the opposite direction was not evaluable as it was too close to the primer sequence. We used two independent methods (PCR-RFLP, TaqMan) for independent confirmation of the variant. As none of the methods reproduced the variant allele we applied bi-directional sequencing with primers up- and downstream of the initial primers. Again, the variant could not be re-detected. Hence we concluded that the variant is a sequencing artifact only.

SULT1A1 MELIQDTSRPPLEYVKGVPLIKYFAEALGPLQSFQARPDDLLISTYPKSG

SULT1A2 MELIQDISRPPLEYVKGVPLIKYFAEALGPLQSFQARPDDLLISTYPKSG

SULT1A3 MELIQDTSRPPLEYVKGVPLIKYFAEALGPLQSFQARPDDLLINTYPKSG

SULT1A4 MELIQDTSRPPLEYVKGVPLIKYFAEALGPLQSFQARPDDLLINTYPKSG

****** ************************************.******

SULT1A1 TTWVSQILDMIYQGGDLEKCHRAPIFMRVPFLEFKAPGIPSGMETLKDTP

SULT1A2 TTWVSQILDMIYQGGDLEKCHRAPIFMRVPFLEFKVPGIPSGMETLKNTP

SULT1A3 TTWVSQILDMIYQGGDLEKCNRAPIYVRVPFLEVNDPGEPSGLETLKDTP

SULT1A4 TTWVSQILDMIYQGGDLEKCNRAPIYVRVPFLEVNDPGEPSGLETLKDTP

********************:****::******.: ** ***:****:**

SULT1A1 APRLLKTHLPLALLPQTLLDQKVKVVYVARNAKDVAVSYYHFYHMAKVHP

SULT1A2 APRLLKTHLPLALLPQTLLDQKVKVVYVARNAKDVAVSYYHFYHMAKVYP

SULT1A3 PPRLIKSHLPLALLPQTLLDQKVKVVYVARNPKDVAVSYYHFHRMEKAHP

SULT1A4 PPRLIKSHLPLALLPQTLLDQKVKVVYVARNPKDVAVSYYHFHRMEKAHP

.***:*:************************.**********::* *.:*

SULT1A1 EPGTWDSFLEKFMVGEVSYGSWYQHVQEWWELSRTHPVLYLFYEDMKENP

SULT1A2 HPGTWESFLEKFMAGEVSYGSWYQHVQEWWELSRTHPVLYLFYEDMKENP

SULT1A3 EPGTWDSFLEKFMAGEVSYGSWYQHVQEWWELSRTHPVLYLFYEDMKENP

SULT1A4 EPGTWDSFLEKFMAGEVSYGSWYQHVQEWWELSRTHPVLYLFYEDMKENP

.****:*******.************************************

SULT1A1 KREIQKILEFVGRSLPEETVDFVVQHTSFKEMKKNPMTNYTTVPQEFMDH

SULT1A2 KREIQKILEFVGRSLPEETVDLMVEHTSFKEMKKNPMTNYTTVRREFMDH

SULT1A3 KREIQKILEFVGRSLPEETMDFMVQHTSFKEMKKNPMTNYTTVPQELMDH

SULT1A4 KREIQKILEFVGRSLPEETMDFMVQHTSFKEMKKNPMTNYTTVPQELMDH

*******************:*::*:****************** :*:***

SULT1A1 SISPFMRKGMAGDWKTTFTVAQNERFDADYAEKMAGCSLSFRSEL

SULT1A2 SISPFMRKGMAGDWKTTFTVAQNERFDADYAKKMAGCSLSFRSEL

SULT1A3 SISPFMRKGMAGDWKTTFTVAQNERFDADYAEKMAGCSLSFRSEL

SULT1A4 SISPFMRKGMAGDWKTTFTVAQNERFDADYAEKMAGCSLSFRSEL

*******************************:*************

**Supplementary Figure 2:** **Multiple sequence alignment of SULT1A1, SULT1A2 SULT1A3 and SULT1A4.** The amino acid sequence of the alpha splice variants of SULT1A1 (ENSP00000378971), SULT1A2 (ENSG00000197165), SULT1A3 (ENSP00000346760) and SULT1A4 (ENSP00000378796) were aligned using the program T-Coffee (http://www.ebi.ac.uk/Tools/msa/tcoffee/). Here, an asterisk below the amino acid alignment marks complete sequence identity, while dots below the sequence mark amino acids that share similar side chains or charges. The positions of missense variants detected in the mutation screens are marked in yellow, green marks the variants that could not be verified with independent methods, red marks the variants rs35728980 and rs1059491 which encode Asn235Thr in both SULT1A1 and SULT1A2, respectively.
