# Supplementary material for: Fine Mapping of a GWAS-Derived Obesity Candidate Region on Chromosome 16p11.2
Source: PLoS One. 2015 May 8;10(5):e0125660. doi: 10.1371/journal.pone.0125660 (PMC4425372; doi:10.1371/journal.pone.0125660)
Supplement: S3 Fig — The first picture shows the whole chromosomal region 16p11.2 is displayed with the genes with non-synonymous variants described in this manuscript marked with vertical lines. The underlying picture shows the obesity association signals of SNPs in the GIANT collective (Speliotes et al. 2010). After the whole chromosomal region for reference, the screened genes are depicted with the detected mutations and MAF in CEU according to dbSNP (http://www.ncbi.nlm.nih.gov/projects/SNP/). Here, the horizontal lines symbolize the introns while exons are marked with bars. Functional domains are also included in the graphs. The positions of the variants are indicated with vertical lines. (DOCX) [file pone.0125660.s008.docx]

**Supplementary figure 4: Mutated positions in the obesity candidate genes of chr16p11.2 and regional overview**

**
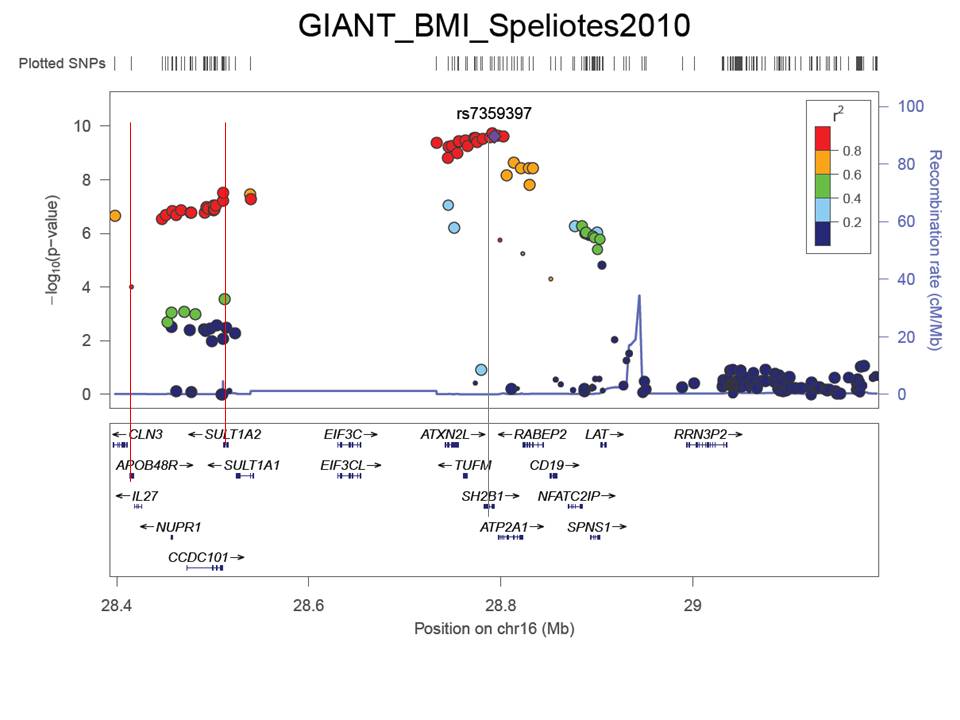
**

**
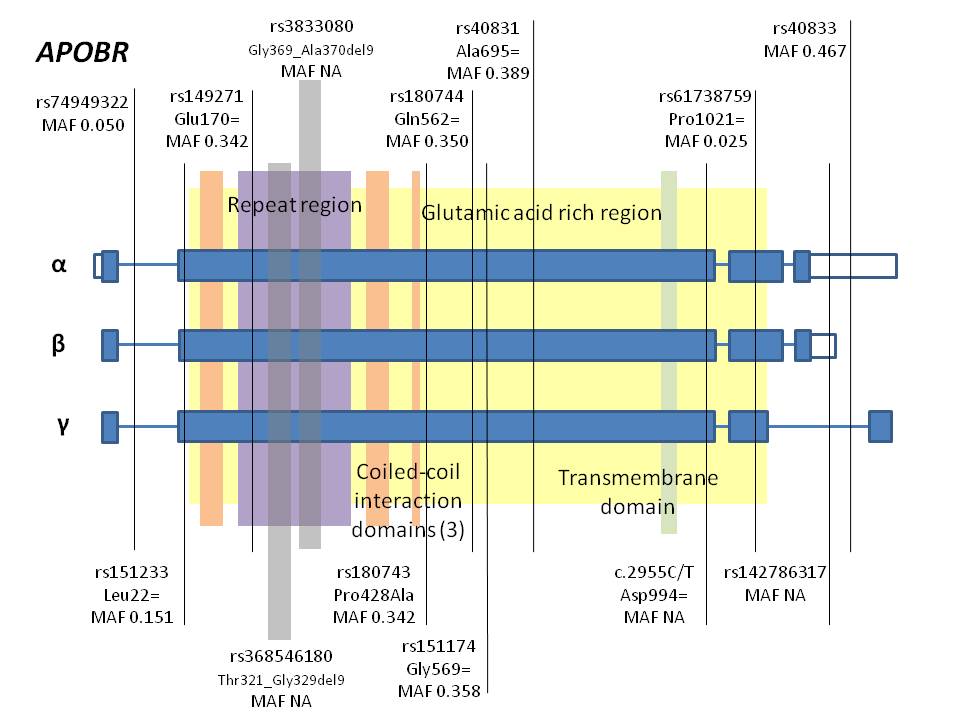

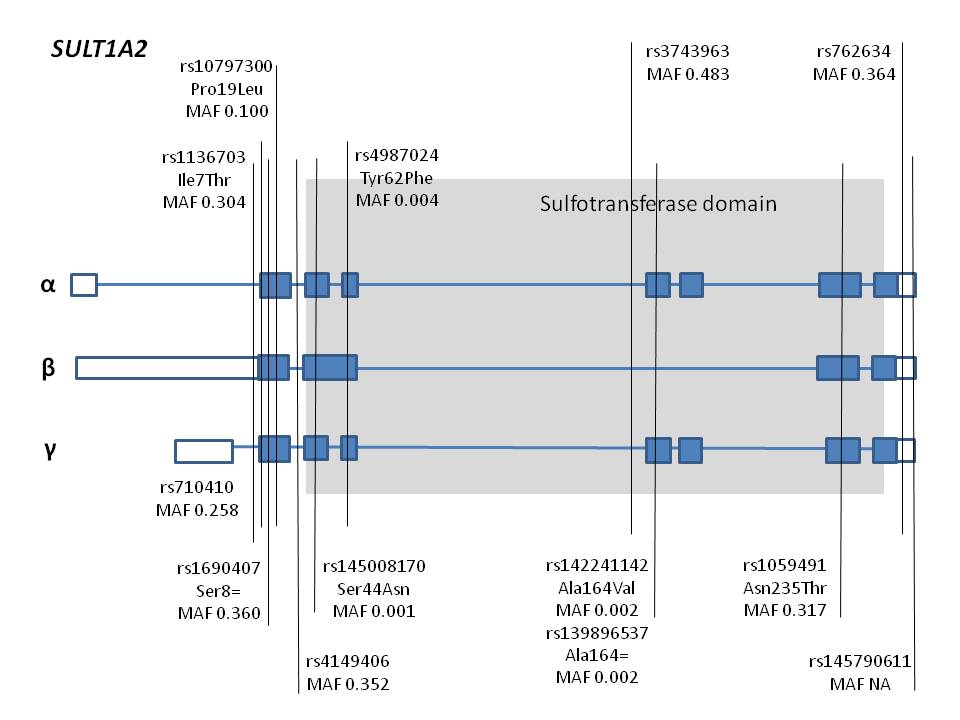
**

**
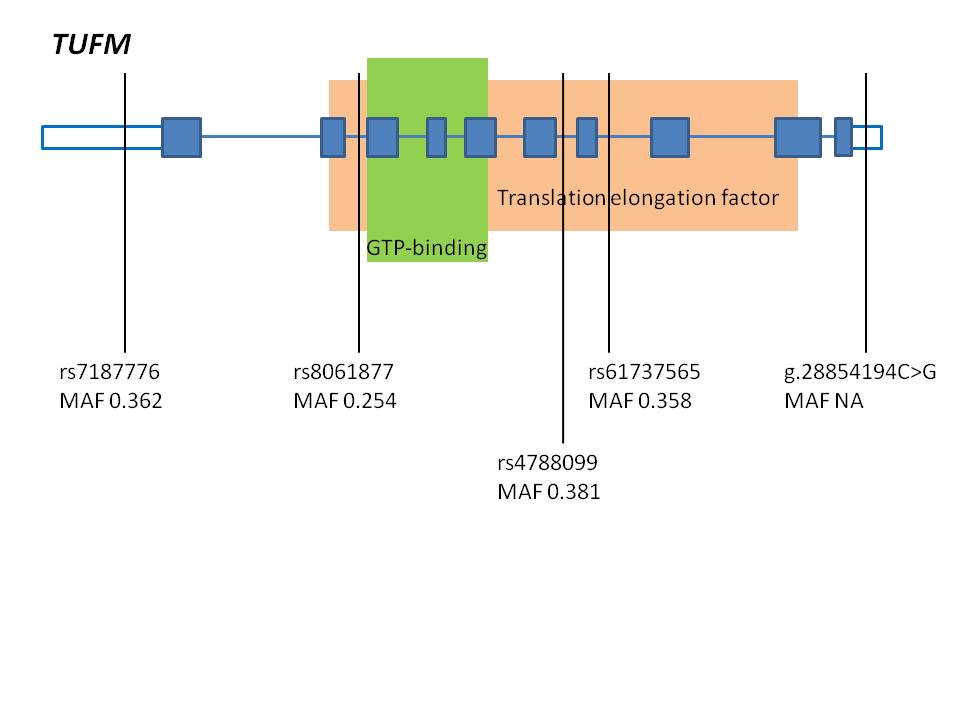
**

The first picture shows the whole chromosomal region 16p11.2 is displayed with the genes with non-synonymous variants described in this manuscript marked with vertical lines. The underlying picture shows the obesity association signals of SNPs in the GIANT collective (Speliotes et al. 2010). After the whole chromosomal region for reference, the screened genes are depicted with the detected mutations and MAF in CEU according to dbSNP (http://www.ncbi.nlm.nih.gov/projects/SNP/). Here, the horizontal lines symbolize the introns while exons are marked with bars. Functional domains are also included in the graphs. The positions of the variants are indicated with vertical lines.
